# PyMulSim: a method for computing node similarities between multilayer networks via graph isomorphism networks

**DOI:** 10.1186/s12859-024-05830-6

**Published:** 2024-06-13

**Authors:** Pietro Cinaglia

**Affiliations:** 1 Department of Health Sciences, Magna Graecia University, Catanzaro, 88100 Italy; 2 Data Analytics Research Center, Magna Graecia University, Catanzaro, 88100 Italy

**Keywords:** Node similarity, Multilayer network, Network analysis, Embeddings, Network alignment

## Abstract

**Background:**

In bioinformatics, interactions are modelled as networks, based on graph models. Generally, these support a single-layer structure which incorporates a specific entity (i.e., node) and only one type of link (i.e., edge). However, real-world biological systems consisting of biological objects belonging to heterogeneous entities, and these operate and influence each other in multiple contexts, simultaneously. Usually, node similarities are investigated to assess the relatedness between biological objects in a network of interest, and node embeddings are widely used for studying novel interaction from a topological point of view. About that, the state-of-the-art presents several methods for evaluating the node similarity inside a given network, but methodologies able to evaluate similarities between pairs of nodes belonging to different networks are missing. The latter are crucial for studies that relate different biological networks, e.g., for Network Alignment or to evaluate the possible evolution of the interactions of a little-known network on the basis of a well-known one. Existing methods are ineffective in evaluating nodes outside their structure, even more so in the context of multilayer networks, in which the topic still exploits approaches adapted from static networks. In this paper, we presented *pyMulSim*, a novel method for computing the pairwise similarities between nodes belonging to different multilayer networks. It uses a Graph Isomorphism Network (GIN) for the representative learning of node features, that uses for processing the embeddings and computing the similarities between the pairs of nodes of different multilayer networks.

**Results:**

Our experimentation investigated the performance of our method. Results show that our method effectively evaluates the similarities between the biological objects of a source multilayer network to a target one, based on the analysis of the node embeddings. Results have been also assessed for different noise levels, also through statistical significance analyses properly performed for this purpose.

**Conclusions:**

*PyMulSim* is a novel method for computing the pairwise similarities between nodes belonging to different multilayer networks, by using a GIN for learning node embeddings. It has been evaluated both in terms of performance and validity, reporting a high degree of reliability.

## Background

Biological systems are comprised of a multitude of individual objects (e.g., molecules, genes, cells, organisms, or entire ecosystems) connected among them. Network theory models biological objects as nodes, while the related interactions (or relationships) as edges. It is a part of graph theory, which defines a network as a graph, where vertices (nodes) and links (edges) possess attributes.

Usually, the terms *graph* and *network* are used interchangeably, however, *graph* tends to be more common in formal areas (e.g., mathematics, computer science) while *network* in fields of a more applicative nature (e.g., biology, bioinformatics). In this paper, we will use *network* to identify the model that represents biological interactions, and *graph* to refer to its formal structure. Similarly, the terms *biological entity* and *biological object* are often used interchangeably, in bioinformatics and related contexts. In detail, an entity refers to any living or non-living component within a biological system, while an object is somewhat more specific and is often used to describe a tangible or well-defined item within a biological system. We will not stick to this subtle differentiation in our paper, conforming to the interchangeability of terminologies.

Biological interactions are generally studied to investigate essential processes, such as: gene regulation, disease propagation, drug effectiveness, evolutionary pathways, and biological interactions [1, 2]. Traditionally, these are represented by using single-layer networks which support an only specific type of entities and interactions (e.g., protein-protein interactions, gene-gene interactions, drug-drug interactions). However, real-world biological systems consisting of biological objects belonging to heterogeneous entities that operate and influence each other in multiple contexts, simultaneously, since any biological function is rarely taken into consideration as an isolated element in the overall system. Therefore, a (typical) static representation on a single layer does not allow evaluating the heterogeneity of the interactions that occur in a biological system, limiting the overall vision.

In this context, multilayer networks are establishing as a novel model for the intricate and heterogenous interactions among (as many heterogeneous) biological objects. These allow representing complex systems as a collection of interconnected layers, referring each one to the biological entity to which a set of objects belongs.

In network science, node similarity represents a particularly relevant research area. Regardless of the network model, nodes with similar features can be clustered in communities, whose internal and external interactions are driven by the effect of *homophily* and *heterophily*. We may denote these as follows: when interactions between nodes belonging to the same cluster occur more often than expected, the network is considered as *homophilic*; otherwise, a network is considered as *heterophilic* when interactions between nodes belonging to different clusters occur more often than expected. As for our interest, it is important to specify that homogeneous biological networks are homophilic, therefore these are driven by the general principle “similarity breeds connections” [3]. However, a multilayer network may represent an exception if its own objects consists of heterogeneous entities, as the heterophily will be essential for the definition of the interlayer edges and cannot be omitted in the similarity analysis; by extension, the same issue concerns heterogeneous networks. It is evident as the evaluation of node similarity is strongly affected by the network model (e.g., static, temporal, heterogeneous, multilayer), as well as the context in which the analysis takes place: within the network or between networks. Briefly, it is enough to understand that a method for evaluating node similarity cannot ignore the model, hence the use of a method for a static model on a multilayer network may lead to misleading results.

In recent years, Graph Neural Network (GNN) [4] have proven to be a relevant tool in research on interaction networks for purposes related to the prediction and classification of graph-based structures, based on node similarity [5]. For instance, GNNs have demonstrated efficiency and robustness in tasks for the identification of candidate gene-disease associations [6], and for inferring drug-target interactions [7] or protein-protein interactions [8], as well as for integrating the close relations between molecular interaction networks and functional genomics data [9].

The most common GNN architectures are: Graph Convolutional Network (GCN) [10], Graph SAmple and aggreGatE (GraphSAGE) [11], Graph Attention Network (GAN) [12], and Graph Isomorphism Network (GIN) [13].

In the context of our interest mainly focused on the topological analysis of a multilayer network and the representation learning for node embedding, the GIN stand out as a compelling choice for several issues, of which we briefly describe the key-points as follows. A GIN offers an end-to-end learning, wherein both node- and graph- level features can be seamlessly integrated and optimized jointly via its own holistic approach, which ensures that relevant information from both local and global structures is effectively captured for a more robust and accurate prediction. All this translates in its ability to capture and leverage topological similarities between node of multiple networks, deeply investigating the structure of the underlying graphs [14]. Unlike traditional architectures that only support simplistic network representations, a GIN offers a data-driven approach that can adapt to the inherent complexity and variability of real-world network data, effectively accommodating to node connectivity and to the size of the network. Furthermore, compared to other architectures, a GIN exhibits remarkable flexibility and expressive power [15]. GIN exhibited high representational power, outperforming other GNNs in terms of accuracy, classification benchmarks, and performance. According to Xu et al. [16], the GIN almost perfectly fits the training data, proving an (empirically) high representational power, while the other GNN architectures severely underfit the training data, in a comparative analysis. This aspect leads to consider the GIN as the most powerful GNN architecture in terms of discriminative power, as well as the most suitable architecture in tasks where discriminating entities in the presence of noise is fundamental [17]. For our purpose, this versatility represents a further advantage, especially if we take into consideration that the scope of our application is biological networks, which by nature exhibit intricate patterns and relationships. From the point of view of scalability as the number of nodes increases, GINs adapt efficiently to large-scale networks in tasks concerning node classification, clustering, and link prediction [18]. Ultimately, we cannot leave it out the incorporation of insights from isomorphism testing [19] enhances the discriminative capabilities of GINs, enabling these to discern subtle structural similarities that might evade traditional methods.

As just described, we have designed the proposed method by incorporating a GIN, as we considered it the most suitable for learning node embeddings from multilayer networks, where nodes belonging to different layers that exist in an interlayer can deviate the similarity calculation, acting as noise.

Node embedding is widely used for exploiting the topological structure within biological networks. For instance, Wilson et al. [20] took advantage of “multi-node2vec” [21] for studying multilayer functional networks modelled from Functional Magnetic Resonance Imaging scans. Their algorithm seeks to maximize the log-likelihood objective function by approximating the continuous node feature representations within a multilayer network. It bases the processing on a second-order random walk sampling procedure that explores multilayer neighbourhoods from the inter- and intra-layer ties. Similarly, Saxena et al. [22] proposed a method for node embedding able to capture both similarities between the nodes and the community structure, while learning the low-dimensional representation of the network.

It is evident how the evaluation of node similarities is a crucial task in network analysis. It is almost always applied within the same network structure, but what has been described so far is also an advantage when applied to the determination of similarities between different networks. In the first case, it allows identifying influential nodes, selecting features of interest, predicting candidate links as well as determining novel interactions or associations, recognizing patterns, detecting overlapping nodes [23]. Between different networks, it is crucial to perform Network Alignment (NA) via node mapping [24], in addition to what has already been described for the other case.

Furthermore, several measures exist to evaluate similarities among nodes within a given network, trivially the analysis of neighbours or its links close to the node of interest [25–27].

This has a completely different relevance when comparing two different networks that do not share links with each other, and whose analyses are carried out independently. To overcome this issue, it is essential to be able to obtain a sort of signature of the node in the source structure, so that this can be evaluated in the target structure. However, the state-of-the-art presents several methods for evaluating the similarity between the nodes of a network, while the cross-network analysis of node similarity in a multilayered topology is missing.

For a more formal definition, without explicitly extending its use to the bioinformatics field, the similarity measure proposed by Mollgaard et al. [28] is worth mentioning. This is a tunable measure for analysing the similarity of nodes across different link weights within a multilayer network. Similarly, Yuvaraj et al. [29] presented a perspective on multilayer network clustering, using the machinery of topological data analysis to group nodes based on how similar their local neighbourhoods are at various resolution scales, without learning or considering the pairwise connectivity patterns or relationships.

As described, methodologies that allow evaluating the similarity between pairs of nodes of different multilayer networks are missing. The latter is crucial in all those methodologies that relate different multilayer networks, e.g., to carry out NA. Existing methods are ineffective in evaluating nodes outside their structure, even more so in the context of multilayer networks, in which the topic still exploits approaches adapted from static networks. It can be seen that, from a careful and in-depth investigation into the state of the art relating to the use of GNNs, or its declinations (e.g., GINs), for comparative purposes between different multilayer networks, at the moment no method or tool is capable of solving this problem.

**Our Contribution** The computation of node similarities plays a relevant role in bioinformatics, being a powerful tool for knowledge extraction from complex biological data, e.g., modelled as a network. In network science, node similarities are investigated to assess the relatedness between biological objects (i.e., nodes), by supporting several tasks in their own development; for instance, these are used in NA, as well as for functional annotation, disease gene prioritization, and other applications having significant implications for understanding of biological systems [30], and more generally, for healthcare [31]. Therefore, the computation of node similarities, and the related measures, allow exploiting information within biological networks, by also supporting the correlated tasks (e.g., NA).

Recently, we proposed *MultiGlobAl* [32] and *Dante* [33], for the pairwise global NA of multilayer networks and dynamic networks, respectively. These build the similarity matrix from two given networks of interest, seeking to globally produce the optimal mapping between their nodes. The similarity are evaluated based on node embedding, that are computed by using an ad-hoc extended version of *Node2Vec* [34]. *Node2Vec* in turn uses the Skip-Gram model of *Word2Vec* [35] based on an Artificial Neural Network (ANN).

However, we had to design and implement several improvements to adapt it in computing node similarity between different networks; it was originally designed (like the others mentioned methods for embedding) to only work within the same network. Briefly, we have extended its design to process information over the layers or time points for multilayer networks or dynamic networks, respectively, by also evolving the original node processing.

On this basis and given the state of the art in the field of node similarity assessment between different networks is not consolidated, and that the topic of multilayer networks is rapidly growing and therefore presents several gaps in this and other issues.

Furthermore, given the existing methods have focused on the evaluation of node similarity within the same network, and that the evaluation of the similarity between nodes of different networks currently represents a gap in literature.

In this paper, we present *pyMulSim*, a method designed specifically for the topic of interest, overcoming the limitations and issues of the method mentioned above. It allows computing the pairwise similarities between nodes belonging to different multilayer networks, based on a GIN for the representative learning of node features. It is able to carry out an analysis of the latter even if they belong to different networks, in order to evaluate their similarity in the form of a similarity matrix.

Our main contribution aims in computing the similarities which allows measuring how much each node in a source multilayer network is similar to the nodes of a target one, allowing also to identify it in a specific layer and, more generally, in a precise region of the latter. These may be modelled as a similarity matrix, or nested lists of pairwise similarities, indifferently. We addressed this problem by using a model based on GIN and the resulting embeddings computed by this one. Briefly, it allows learning low-dimensional embeddings of nodes both in source and target networks, for subsequent similarity computation.

## Materials and methods

### Problem definition

The key-issue addressed in our work concerns how much each node in a source multilayer network is similar to a node of a target one, maintaining the layered structure in which these may coexist. More exhaustively, the analysis has to occur layer-on-layer in that heterogeneous entities must not be mixed in processing. This last issue was in fact considered in our experimentation as the occurrence of a *de facto* false positive. Briefly, nodes belonging to different entities are different by nature, as the context of interest concerns the biological networks; to give a non-exhaustive but nevertheless explanatory example: a gene cannot therefore be similar in any way to a drug or a disease, beyond the relationship (e.g., interaction, association) that it has with this.

Before proceeding with the formal definition of the problem, we briefly introduce the basic notations that we will use.

Formally, a multilayer network can be described as $G_M = (V_M, E_M)$, where $V_M$ and $E_M$ are a set of nodes and edges, respectively [36].

Referring to $G_{M}$, let us denote a generic intralayer by $G_{a}$ and a generic interlayer by $G_{b}$, consisting of its own set of edges $E_a$ (i.e., intralayer edges) and $E_b$ (i.e., interlayer edges), respectively:

1 $$\begin{aligned} G_{a}= & {} (V_M, E_a): E_a = {((u, \alpha ), (v, \beta )) \in E_M | \alpha = \beta } \end{aligned}$$

2 $$\begin{aligned} G_{b}= & {} (V_M, E_b): E_b = E_M / E_a \end{aligned}$$

with $\alpha$ and $\beta$ the arrays of elementary layers, and (*u*, *v*) a generic pair of nodes. Note that in the proposed model, edges are undirected.

For a given multilayer network, its input data consists of a set of tuples (*u*, *v*, *l*) (i.e., edges list with attributes), where *u* and *v* are two nodes affecting the edge of interest, and *l* is the layer identifier on which the latter insists. Note that layer identifiers are also used to discriminate intralayers and interlayers edges, and these must be provided as input; alternatively, a further attribute may be implemented to report the type of edge (e.g., $\{id:l, edge\_type:intralayer|interlayer\}$).

**Fig. 1:**
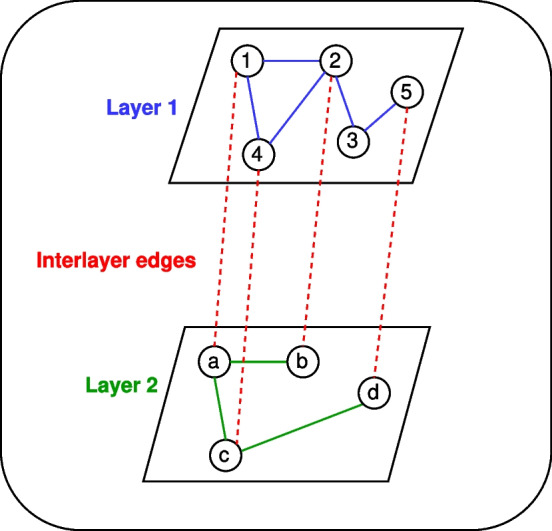
A non-exhaustive example for a (toy) multilayer network consisting of two layers. The blue edges (i.e., intralayer edges) refer to the first layer, while the green edges (i.e., intralayer edges) to the second layer. Interlayer edges are depicted in red

Figure 1 shows a non-exhaustive example for a (toy) multilayer network, for illustrative purpose only.

Based on this formulation, we describe the problem definition below.

Let us denote two multilayer networks as $G_1 = (V_1, E_1)$ and $G_2 = (V_2, E_2)$, with *n* layers; $G_1$ and $G_2$ must have the same number of layers (*n*).

The task consists in evaluating the node similarity between $G_1$ and $G_2$, across the same layers. First, it involves computing node embeddings layer-on-layer, as vector of vectors (or matrix). Subsequently, it measures the similarity between corresponding node embeddings in each one, by using a proximity measurement.

Let $\textbf{X}_1$ and $\textbf{X}_2$ be the node embedding matrices for $G_1$ and $G_2$, respectively, each row of $\textbf{X}_1$ and $\textbf{X}_2$ corresponds to the embedding of a node in the respective network, with $|\textbf{X}_1| = |\textbf{X}_2| = |V|$.

### Graph isomorphism network model

In this section, we explain the key components of our GIN’s model implementation.

Firstly, we formally describe the three main steps on the basis of which a GIN carries out its own processing: Initialization, Aggregation, and Embedding.

**Initialization** (Eq. 3): each node, in the input network *G*, is assigned an initial feature vector.

3 $$\begin{aligned} \forall v \in V: h_v^{(0)} = InitialFeatures(v) \end{aligned}$$

where *InitialFeatures* is a set of possible relevant information (i.e., features) associated to nodes initially given to the model. To give an example referred to a generic gene, it may include: type, regulation, other biological or topological properties, only such as the nearest and most relevant neighbours.

**Aggregation** (Eq. 4): information is aggregated from neighbouring nodes, and the model computes a novel representation for each node. This step is performed for *K* (predefined) iterations, to allow nodes to gather and propagate information from their local neighbourhoods.

4 $$\begin{aligned} h_v^{(k)} = \text {MLP}^{(k)}\left( \left( 1 + \epsilon ^{(k)}\right) \cdot h_v^{(k-1)} + \sum _{u \in N(v)} h_u^{(k-1)}\right) \end{aligned}$$

where $h_v^{(k)}$ is the representation of node *v* at *k*-th iteration ($1 \le k \le K$), $\text {MLP}^{(k)}$ is the MultiLayer Perceptron (MLP), $\epsilon ^{(k)}$ and is a learnable parameter. MLP is an ANN, organized in at least three layers, consisting of fully connected (linear) neurons with a nonlinear activation function.

**Node Embeddings** (Eq. 5): a low-dimensional vector representations (i.e., embeddings) of each node is computed by iteratively aggregating topological information through exploration of each node and its neighbours. Specifically, embeddings are taken from the last aggregation step ($k=K$).

5 $$\begin{aligned} h_v^{(\text {final})} = h_v^{(K)} \end{aligned}$$

Therefore, $h_v^{(\text {final})}$ is the learned feature vectors (i.e., node embeddings) for each node in the network, after multiple aggregation steps. Each embedding consists of the information from the node’s local neighbourhood, as well as the overall network structure.

The resulting output is a vector consisting of vector embeddings referred to the nodes from the given network.

Summarizing what has been described so far, the GIN applied in our method computes node embeddings, by iteratively aggregating information from neighbouring nodes, in accordance with the typical GIN architectures foreseen for such uses. The node embeddings represent the characterizations of the related node of interest, having been built on structural and contextual information in the graph.

We implemented the GIN’s layer through PyTorch Geometric for inheriting the graph neural network layers and message (i.e., features) passing; note that the features pass from one node to its neighbours without modification. It performs message aggregation in a layer via element-wise addition.

For each layer, based on MLP architecture (see Sect. 2.2), we used the Rectified Linear Unit (ReLU) as activation function.

Formally, *ReLU* can be defined as follows:

$$ReLU(x) = max(0, x)$$

It is used within MLP for producing node embeddings during the forward pass. Briefly, the latter applies the neural network to the input features and then passes the transformed information to neighbouring nodes. ReLU was applied between the two fully-connected (linear) layers of MLP.

Multiple instances of our layer were used to model the entire GIN; usually, the latter has at least three layers. Our task is limited to compute node embeddings, and it can be considered the first set of operations that are typically performed for more complex issues such as classification and prediction; consequently we limited ourselves to a rather simple model, in terms of number of layers, which therefore does not show unnecessary overloads of components. Therefore, our GIN’s model consists of an initial layer (*conv1*), multiple intermediate layers (*convs*), and a final layer (*conv2*). The model takes the input multilayer network and sequentially passes it through the layers. The intermediate layers (i.e., *convs*) are defined in accordance with the number of layers existing within the multilayer network of interest.

Our implementation defines a GIN for processing multilayer networks, and more generally, graph-structured data. Layers are the fundamental components that perform information propagation and transformation, and the GIN class combines these layers to create a complete neural network for graph-related tasks.

**Fig. 2:**
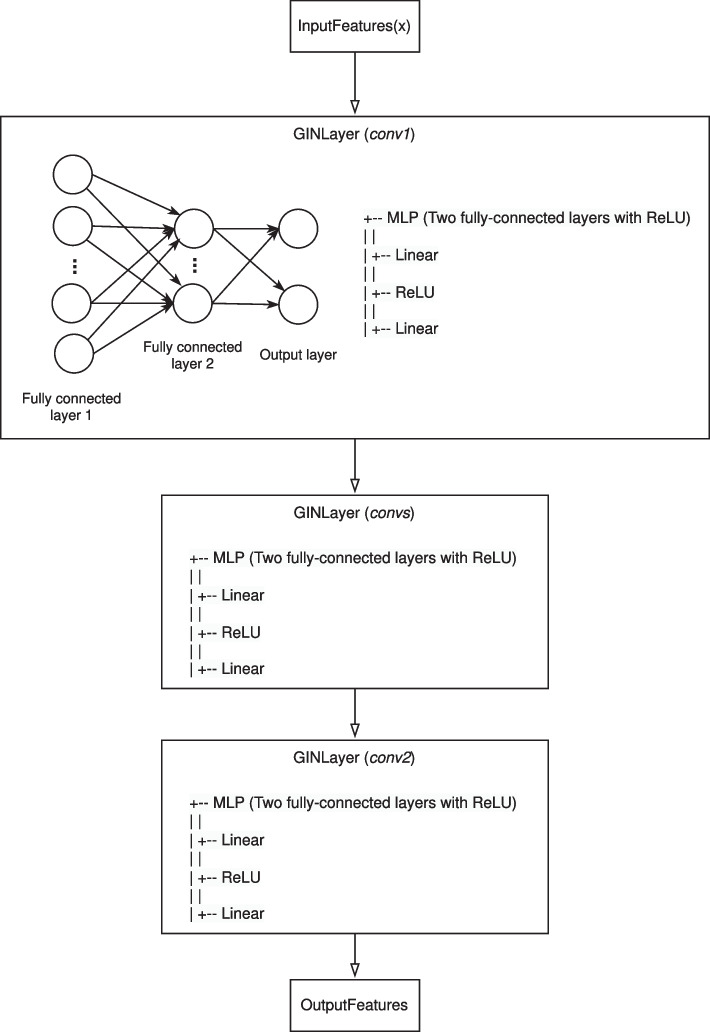
We depicted the architecture of the GIN in *pyMulSim*, for illustrative purpose only. In terms of flow of data, it starts with input features (*x*) and passes through multiple layers (*GINLayer*): *conv1*, *convs*, and *conv2*, consisting of an MLP having two fully-connected (linear) layers and ReLU as activation function. Embeddings are output from the last layer (*conv2*)

Fig. 2 shows a non-exhaustive representation of the GIN architecture used by *pyMulSim*.

### Preprocessing

The multilayer networks of interest are preprocessed to convert their own information to a structure learnable by our GIN, passing by NetworkX [37]. Specifically, we carry data from a NetworkX object to a PyTorch Geometric’s *Data* one [38].

NetworkX does not support multilayer networks, therefore, we defined a multilayer network as a set of graphs where each one represent a layer, in order to import the described flat representation. Briefly, we dismember the initial structure so as not to lose information and reuse it in the next step. Eventually, the multilayer networks can be directly passed in this format, skipping reading from the file system.

We describe below the main steps of preprocessing, before introducing the preliminary data import and parsing, for completeness.

**Data import and parsing** It trivially reads the input data, specifically, two given multilayer networks stored in accordance with the model described in Sect. 2.1. Each network is imported as a *NetworkX* object, which creates a graph structure containing edges and associated data, for each layer. Our implementation is able to include information about the layer to which each edge belongs, in order to extend the support from simple networks to multilayer ones.

**Preprocessing** It is the core of what is described in the current section, and we can simplify its processing as follows:

- Each multilayer network in input is divided into its own individual layers, by keeping the reference to the interlayer edges, in order to evaluate the links between layers. Each layer is structured as a subgraph, to facilitate further processing.
- The function handles interlayer edges, which connect nodes between adjacent layers. These interlayer edges are added to both the current layer and the next layer to model relationships between nodes in different layers.
- Node features are investigated for each node, of each layer. The initial features are provided based on the nearest neighbours that each node reports in its topology.
- Node features are assigned to all nodes, including those involved in interlayer edges. The number of features assigned to each node is determined by the input channels parameter (default: 64).
- The graph data for all layers is converted in PyTorch Geometric’s Data object.

Ultimately, the resulting data object includes information about node features, edge indices, and interlayer edges, by making the data suitable for several deep learning models, including obviously our own GIN. This approach allowed us to describe heterogeneous graphs, holding multiple node and edge types in disjunct storage objects (layers).

At low-level, the data structure used in our implementation is a tensor, i.e., a multidimensional matrix containing elements of a single data type. A tensor can be constructed from a list or sequence of objects, just as we did by dismembering and reconstructing the elements of the multilayer network, without loss of information.

### Embedding-based node similarity

Nodes that are structurally similar may end up with similar final embeddings. The behaviour of a given biological object, analysed on the basis of interactions, is indicative of its biological functionality, the latter being directly related to the type and modalities of the interactions that insist on it or that propagate from it.

For node-level tasks, we can directly use the node embeddings for classification, regression, or others, without the need for a global aggregation. Therefore, the proposed method uses these for computing node similarities, directly. To give an example, common aggregation operations can include sum, mean, or max pooling over the node embeddings.

Firstly, our GIN (see Sect. 2.2) is used to produce the embeddings of each node, for the two multilayer networks of interest, based on preprocessed data (see Sect. 2.3); therefore, the latter is integrated with node embeddings.

For evaluating the similarities, we used the Jaccard’s similarity coefficient (Jc) [39], a well-known proximity measurement in the field of node similarity applied to network science.

Jc is formally described in Eq. 6.

6 $$\begin{aligned} Jc = \frac{|\Gamma (u) \cap \Gamma (v)|}{|\Gamma (u) \cup \Gamma (v)|} \end{aligned}$$

with *u* and *v* the two nodes of interest, and $\Gamma (u)$ the set of neighbours of *u*.

Jc assesses the Intersection-over-Union between the neighbours of two nodes by normalizing the size of the intersection by the size of the union. Therefore, the resulting similarity will be a value between 0 and 1, representing $0\%$ and $100\%$ of similarity, respectively, between two nodes of interest.

The choice to use Jc was motivated by a comparative evaluation carried out within the study proposed by CoÅŸkun et al. [40]. The latter reported Jc as an appreciable asymmetric measure in terms of effectiveness, compared to well-known ones for evaluating the node similarity in biological networks by applying graph representation learning methods, such as: Common Neighbors, Adamic-Adar, ResourceAllocation, Hub-Depressed Index, Hub-Promoted Index, SÃ¸renson Index. Generally, Jc has been proved to be a proper metric by also satisfying crucial properties (e.g., equivalence closure, symmetry and triangle inequality), furthermore it has the advantage of being an asymmetric measure that goes well with the multilayer networks of our interest, as these are undirected [41, 42].

## Experimentation

In this section, we report the main information about our experimentation, as well as the related results both on synthetic and real datasets.

### Datasets

Our own dataset consists of 60 multilayer networks, constructed from the Multiplex Network of Human Diseases (MNHD) provided by Halu et al. [43].

MNHD has been constructed by projecting the bipartite networks of the Disease-Symptom interactions and Gene-Disease interactions. Specifically, two datasets from Genome-wide Association Study (GWAS) and Online Mendelian Inheritance in Man (OMIM) catalogues, respectively, are released. These are almost equivalent even if based on different identifiers, therefore we arbitrarily chose the one with OMIM identifiers. MNHD consists of two layers: Gene-Gene interactions and Disease-Disease interactions, respectively; the interlayer edges are retrieved from the original bipartite networks.

Let us denote with *m* the number of (intralayer) edges with which a new node attaches to existing nodes, with *p* the probability that *m* new edges are added, with *q* the probability that *m* existing edges are rewired to a random chosen edge and the related node; and with *z* the percentage of (interlayer) edges that are randomly created between two layers.

Zhong et al. [44] investigated generative parameters for biological networks in their own experimentation, suggesting that $n=50$, $m=2$, $p=0.5$, and $q=0.4$ allow preventing the formation of high-density clusters that distort the original conformation of the network, as well as maintaining the interaction between the nodes as similar as possible to the real biological case. Therefore, we obtained the first 10 multilayer networks by applying the reported parameters on MNHD, by applying the methodology designed for *Generator of Interconnected Networks* [45], a software tool for constructing datasets of multilayer networks for bioinformatics.

Furthermore, to aggregate pairs of multilayer networks in samples consisting of known similarities and mappings for reliable and relevant results, we constructed our dataset by replicating the initial networks through the application of noise to the original topology. Specifically, we generated 5 noisy versions for each subnetwork by shuffling the $5\%$, $10\%$, $15\%$, $20\%$, and $25\%$ of the whole set of intralayer and interlayer edges, randomly.

The resulting dataset consisted of 60 multilayer networks from real-world biological data. Samples have been formed by pairing each initial multilayer subnetwork with its noisy counterparts.

In our experimentation, we refer to *Ground Truth* as the information that is known to be real or true, provided by direct measurement and empirical evidence. Dataset construction is performed from real-world data, in accordance with Halu et al. [43], by randomly extracting a set of subnetworks to increase the overall size for testing purposes, therefore the real/true similarity between the nodes of the source and target, respectively, is known for empirical evidence. We also tested our method in a real-use case by computing the similarity matrices on which performing NA between the pairs in each sample. As described, the latter consist of a pair of multilayer networks in which one is the noisy counterpart of the other, therefore the node mapping is known since both ones contain the same set of nodes on the same layers. In this specific case, the Ground Truth was the True Node Mapping between the nodes of the source network and their counterparts in the target network.

### Results

We evaluated our method by using the following well-known Key Performance Indicator (KPI): accuracy, AUC, sensitivity, specificity, precision, F1-score. We also evaluate the performance in respect to the noise levels. Results are reported in Tables 1, 2, and 3. The latter (i.e., Wald test) is a non-parametric alternative to the t-test, which we did anyway (see Table 6).

In addition, we evaluated the node similarities computed by the proposed method, statistically, in order to corroborate its own validity and effectiveness (see Tables 4, 5, and 6).

Finally, we also tested our method in a real-use case related to NA, in terms of Node Correctness (NC). We aligned each (initial) multilayer network with its own noisy versions, from our dataset. This test was made possible by applying the *MultiGlobAl*’s objective function to node similarities computed by *pyMulSim*. Results have been normalized for an effective representation and interpretation, for both methods.

Alignments (i.e., node mappings) were obtained by applying the objective function of *MultiGlobAl* to the node similarities computed by *pyMulSim*. The results were compared with the ones performed by *MultiGlobAl* via its own in-house method based on *node2vec*.

**Fig. 3:**
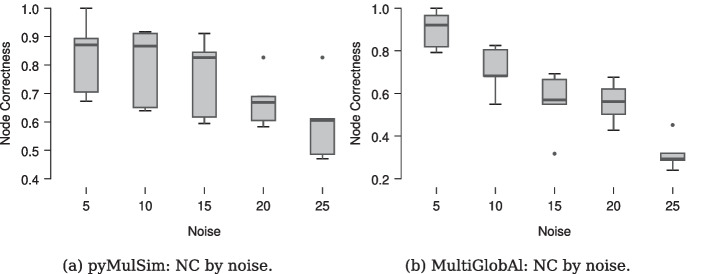
NC computed for the alignments between each (initial) multilayer network and its own noisy versions, by using *MultiGlobAl* and its own objective function applied to node similarities computed by *pyMulSim*. Descriptive statistics has been reported in Table 7

Table 7 shows the descriptive statistics for the alignment performance (i.e., node mappings), in terms of NC, produced by *MultiGlobAl* and its own objective function applied to node similarities from *pyMulSim*. Furthermore, these are shown in Fig. 3.

**Table 1:** The table reports the Key Performance Indicators (i.e., accuracy, AUC, sensitivity, specificity, precision, F1-score), from our experimentation

Accuracy	0.998
AUC	0.954
Sensitivity	0.690
Specificity	0.999
Precision	0.732
F1-score	0.710

**Table 2:** The table reports the confusion matrix from the overall classification of node similarities computed by *pyMulSim* in respect to the ones from Ground Truth

	Predicted		
Observed	1	% Correct	
0	356698	269	99.925
1	330	735	69.014
Overall % Correct			99.833

**Table 3:** The table reports the results from the correct classification of node similarities computed by *pyMulSim* in respect to the ones from Ground Truth

Noise level	Estimate	SE	z	Wald Statistic	df	p
5	$-2.236$	0.103	$-21.610$	467.000	1	$<0.001$
10	$-3.304$	0.144	$-22.889$	523.902	1	$<0.001$
15	$-3.741$	0.179	$-20.890$	436.400	1	$<0.001$
20	$-3.685$	0.191	$-19.296$	372.354	1	$<0.001$
25	$-3.729$	0.217	$-17.169$	294.771	1	$<0.001$

Data are shown by noise level

SE: Standard Error of the Mean, df: Degrees of freedom

**Table 4:** The table reports the Student’s t-test between the similarities computed by *pyMulSim* and Ground Truth

Measure 1		Measure 2	t	df	p
pyMulSim similarities	–	Ground Truth	$-617.749$	358031	$<0.001$

Student’s t-test. df: Degrees of freedom

**Table 5:** The table reports the descriptive statistics related to the results from the Student’s t-test (see Table 4)

	N	Mean	SD	SE	C
pyMulSim	358032	0.008	0.075	$1.252\times 10^{-4}$	8.883
Ground Truth	358032	0.250	0.239	$3.994\times 10^{-4}$	0.955

N: Number of samples, SD: Standard Deviation, SE: Standard Error of the Mean, C: Coefficient of variation

**Table 6:** The table reports the results from the Wilcoxon Signed-Rank test, between the correct classification of a similarity by *pyMulSim*, in respect to the data from Ground Truth

Measure 1		Measure 2	W	z	p
pyMulSim	-	Ground Truth	180953.500	$-52.111$	$<0.001$

The test concerned a use-case related to a binary classification task

Wilcoxon Signed-Rank test

**Table 7:** Real Use-Case. Descriptive Statistics for NC, from alignments (i.e., node mappings) computed by *MultiGlobAl* and *pyMulSim*; the latter inherits the objective function of the former

	Noise	Mean	SD	Minimum	Maximum
pyMulSim	5	0.828	0.137	0.672	1.000
	10	0.797	0.140	0.639	0.917
	15	0.759	0.143	0.594	0.911
	20	0.675	0.096	0.583	0.826
	25	0.600	0.142	0.471	0.826
MultiGlobAl	5	0.899	0.091	0.792	1.000
	10	0.709	0.111	0.550	0.825
	15	0.559	0.148	0.318	0.692
	20	0.558	0.097	0.428	0.676
	25	0.319	0.080	0.240	0.452

SD: Standard Deviation

## Discussion

In this section, we discuss the results reported in Sect. 3.

However, let us first introduce some preliminary aspects, relevant to understanding the choices made for the experimentation. Foremost, we report below the reasons for exclusions of the solutions mentioned in the previous sections. The main limitation that did not allow us to include *multi-node2vec* [21] in our experimentation was the possibility of evaluating the similarities between nodes within the same network, without lending itself to pairwise similarity between different networks. The same issue concerned *NodeSim*, which anyway does not support multilayer networks. Furthermore, the similarity measure presented by Mollgaard et al. [28] is a formal definition used for the sole purpose of testing its validity; no implementation has been released, as well as any ready-to-use software tools. No methods specifically developed for the presented purpose are currently available.

We evaluated the performance of our solution by using several well-known KPI. Table 1 and the related Table 2 show that *pyMulSim* effectively calculate the similarities between the biological objects of a multilayer network by using the features from the embeddings, computed by its own GIN.

The interpretation of the KPI of interest (i.e., accuracy, auc, sensitivity, specificity, precision, and F1-score) shows an overall good performance [46, 47], by corroborating the goodness in determining the similarities between pairs of nodes of different networks, as well as an outstanding discrimination among node pairs (see AUC).

These capabilities are also maintained at different noise levels, i.e., $5\%$, $10\%$, $15\%$, $20\%$, and $25\%$. Therefore, the Wald test [48] shows a strong statistical significance ($p<0.001$). The latter is a non-parametric alternative to the t-test, which we did anyway (see Table 6).

For statistical assessment, we performed a student’s t-test [49] to analyses the statistical significance of pairwise similarities computed by *pyMulSim*, in respect to Ground Truth.

We further corroborated this issue by conducing a binary classification task, in which a similarity can be considered as correctly identified (i.e., true, or 1), or no (i.e., false, or 0); the benchmarks of this analysis are evaluated in respect to the Ground Truth. Specifically, we applied a threshold equal to 0.95: the similarity between two nodes is correctly recognized, if and only if it falls within a $95\%$ confidence interval (as per conventional criteria). Data were analysed by using the Wilcoxon Signed-Rank test [50], a non-parametric statistical hypothesis test which allows us to assess the correct identification of a similarity in a binary classification task, as described.

By conventional criteria, the results (see Table 4 and Table 6) proved to be in line with expectations, reporting an actual statistical significance ($p<0.001$), which demonstrated how the similarities obtained from our solutions are to be considered valid and effective. The coefficient of variation of *pyMulSim* (see Table 5) was found to be higher than one processed from the Ground Truth. The latter is intrinsic to the way our method operates: it is obviously not able to detect any biological or homological similarity among objects that do not share substructures or interactions, its analysis being exclusively performed from a topological point of view.

Finally, we applied *pyMulSim* to a real-use cases related to NA. This test shows that node similarities computed by *pyMulSim* allows improving by an average of $20.185\%$ (see Table 7), the alignments (i.e., node mappings) in terms of NC. Furthermore, this test also shows a better noise tolerance compared to the comparison methodology.

## Conclusions

The network’s interactions allow representing essential functional processes of a real-world biological system. However, the latter evolve through multiple layers, which may represent different types of relationships or entities, as well as different temporal points of its evolution. In network science, node embeddings are widely used for exploiting the topological structure within biological networks, and deep learning techniques and models are used for their own representative learning. In this paper, we presented *pyMulSim*, a novel method for computing pairwise similarities between nodes belonging to different multilayer networks (source and target, respectively), exploiting the embeddings computed through a GIN architecture. We investigated the performance of our method in an in-depth and dedicated experimentation, where *pyMulSim* showed a high degree of reliability. Statistical assessment and the performance evaluation effectively corroborate the validity of resulting node similarities. In addition, the tests have been conducted on several noise levels, in source and target topologies. The results proved to be statistically significant, demonstrating the goodness of the data (i.e., the similarities of the nodes) calculated by our method.

Finally, *pyMulSim* has been used to address a real-use case, concerning the NA. In this test, it showed its own node similarities can improve NA between multilayer networks by an average of $20.185\%$ (Table 7), in terms of NC, by also showing a good robustness in the presence of noise.

The main noteworthy limitation found in our experimentation is intrinsic to the topological similarity approach itself: our method is obviously not able to detect any biological or homological similarity among objects that do not share substructures or interactions, its analysis being exclusively performed from a topological point of view. About this, in hypothetical future works, we do not rule out extending support to other similarity coefficients, also incorporating homological measures.

### Availability and requirements

Project name: pyMulSim. Project home page: https://github.com/pietrocinaglia/pymulsim (accessed on 02 May 2024). Operating system(s): Platform independent. Programming language: Python 3. Other requirements: https://github.com/pietrocinaglia/pymulsim/blob/main/requirements.txt (accessed on 02 May 2024). Licence: the software is provided *AS IS*, under MIT Licence. Any restrictions to use by non-academics: none.

## Data Availability

Data and materials related to *pyMulSim* are available at https://github.com/pietrocinaglia/pymulsim (accessed on 02 May 2024).

## Abbreviations

GIN: Graph Isomorphism Network
GNN: Graph Neural Network
GCN: Graph Convolutional Network
GraphSAGE: Graph SAmple and aggreGatE
GAN: Graph Attention Networks
NA: Network Alignment
ANN: Artificial Neural Network
MLP: MultiLayer Perceptron (MLP)
ReLU: Rectified Linear Unit
MNHD: Multiplex Network of Human Diseases
GWAS: Genome-wide Association Study
OMIM: Online Mendelian Inheritance in Man
KPI: Key Performance Indicator

## References

[1] Cinaglia P, Guzzi PH, Veltri P. Integro: an algorithm for data-integration and disease-gene association. In: 2018 IEEE international conference on bioinformatics and biomedicine (BIBM); 2018. p. 2076–2081 10.1109/BIBM.2018.8621193

[2] Milano M, Cinaglia P, Guzzi PH, Cannataro M (2023). Aligning cross-species interactomes for studying complex and chronic diseases. Life.

[3] Apollonio N, Blankenberg D, Cumbo F, Franciosa PG, Santoni D. Evaluating homophily in networks via HONTO (HOmophily network TOol): a case study of chromosomal interactions in human PPI networks. Bioinformatics. 2023; 39(1) 10.1093/bioinformatics/btac763 PMC9805585 36440918

[4] Wu Z, Pan S, Chen F, Long G, Zhang C, Yu PS (2021). A comprehensive survey on graph neural networks. IEEE Trans Neural Netw Learn Syst.

[5] Yang H, Zhuang Z, Pan W (2021). A graph convolutional neural network for gene expression data analysis with multiple gene networks. Stat Med.

[6] Cinaglia P, Cannataro M. Identifying candidate gene-disease associations via graph neural networks. Entropy (Basel). 2023; 25(6) 10.3390/e25060909 PMC10296901 37372253

[7] Zhang Z, Chen L, Zhong F, Wang D, Jiang J, Zhang S, Jiang H, Zheng M, Li X (2022). Graph neural network approaches for drug-target interactions. Curr Opin Struct Biol.

[8] Wan X, Wu X, Wang D, Tan X, Liu X, Fu Z, Jiang H, Zheng M, Li X. An inductive graph neural network model for compound-protein interaction prediction based on a homogeneous graph. Brief Bioinform. 2022; 23(3) 10.1093/bib/bbac073 PMC9310259 35275993

[9] Hasibi R, Michoel T (2021). A graph feature Auto-Encoder for the prediction of unobserved node features on biological networks. BMC Bioinf.

[10] Kipf TN, Welling M. Semi-supervised classification with graph convolutional networks; 2017

[11] Hamilton WL, Ying R, Leskovec J. Inductive representation learning on large graphs; 2018

[12] Veličković P, Cucurull G, Casanova A, Romero A, Liò P, Bengio Y. Graph attention networks; 2018

[13] Xu K, Li C, Tian Y, Sonobe T, Kawarabayashi K-i, Jegelka S. Representation learning on graphs with jumping knowledge networks. In: Dy J, Krause A (eds) Proceedings of the 35th international conference on machine learning. Proceedings of machine learning research, vol. 80. PMLR; 2018. p. 5453–5462

[14] Xiao J, Yang L, Wang S (2024). Graph isomorphism network for materials property prediction along with explainability analysis. Comput Mater Sci.

[15] Wein S, Schüller A, Tomé AM, Malloni WM, Greenlee MW, Lang EW (2022). Forecasting brain activity based on models of spatiotemporal brain dynamics: a comparison of graph neural network architectures. Netw Neurosci.

[16] Xu K, Hu W, Leskovec J, Jegelka S. How powerful are graph neural networks? In: International conference on learning representations; 2019. https://openreview.net/forum?id=ryGs6iA5Km

[17] Kim B-H, Ye JC (2020). Understanding graph isomorphism network for rs-fMRI functional connectivity analysis. Front Neurosci.

[18] Zheng K, Zhao H, Zhao Q, Wang B, Gao X, Wang J. NASMDR: a framework for miRNA-drug resistance prediction using efficient neural architecture search and graph isomorphism networks. Brief Bioinform. 2022; 23(5) 10.1093/bib/bbac338 35998922

[19] Chen Z, Villar S, Chen L, Bruna J. On the equivalence between graph isomorphism testing and function approximation with GNNS. In: Wallach H, Larochelle H, Beygelzimer A, Alché-Buc F, Fox E, Garnett R (eds) Advances in neural information processing systems, vol. 32. Curran Associates Inc; 2019. p. 1–9

[20] Wilson JD, Baybay M, Sankar R, Stillman P, Popa AM (2021). Analysis of population functional connectivity data via multilayer network embeddings. Netw Sci.

[21] Wilson JD, Baybay M, Sankar R, Stillman PE. Fast embedding of multilayer networks: an algorithm and application to group fmri; 2018. arXiv:abs/1809.06437

[22] Saxena A, Fletcher G, Pechenizkiy M (2022). NodeSim: node similarity based network embedding for diverse link prediction. EPJ Data Sci.

[23] Aleskerov F, Shvydun S (2019). Stability and similarity in networks based on topology and nodes importance. Studies in computational intelligence. Studies in computational intelligence.

[24] Cinaglia P, Cannataro M (2022). Network alignment and motif discovery in dynamic networks. Netw Model Anal Health Inf Bioinf.

[25] Abu-Aisheh Z, Raveaux R, Ramel J-Y, Martineau P. An exact graph edit distance algorithm for solving pattern recognition problems. In: Proceedings of the international conference on pattern recognition applications and methods. SCITEPRESS - Science and and Technology Publications; 2015

[26] Zhang J, Tang J, Ma C, Tong H, Jing Y, Li J. Panther: fast top-k similarity search on large networks. In: Proceedings of the 21th ACM SIGKDD international conference on knowledge discovery and data mining. ACM, New York; 2015

[27] Jeh G, Widom J. SimRank. In: Proceedings of the 8th ACM SIGKDD international conference on knowledge discovery and data mining. New York: ACM; 2002

[28] Mollgaard A, Zettler I, Dammeyer J, Jensen MH, Lehmann S, Mathiesen J (2016). Measure of node similarity in multilayer networks. PLoS ONE.

[29] Yuvaraj M, Dey AK, Lyubchich V, Gel YR, Poor HV (2021). Topological clustering of multilayer networks. Proc Natl Acad Sci USA.

[30] Xu Y, Rockmore D. Feature selection for link prediction. In: Proceedings of the 5th Ph.D. workshop on information and knowledge. PIKM’12. New York: Association for Computing Machinery; 2012. p. 25–32. 10.1145/2389686.2389692

[31] Li Y, Luo P, Wu C. A new network node similarity measure method and its applications; 2014. arXiv:abs/1403.4303

[32] Cinaglia P, Cannataro M (2023). Multiglobal: global alignment of multilayer networks. SoftwareX.

[33] Cinaglia P, Cannataro M (2023). A method based on temporal embedding for the pairwise alignment of dynamic networks. Entropy.

[34] Grover A, Leskovec J (2016). node2vec: scalable feature learning for networks. KDD.

[35] Mikolov T, Sutskever I, Chen K, Corrado G, Dean J. Distributed representations of words and phrases and their compositionality. In: Proceedings of the 26th international conference on neural information processing systems. NIPS’13, vol 2. Curran Associates Inc; 2013. p. 3111–3119

[36] Cinaglia P, Milano M, Cannataro M (2023). Multilayer network alignment based on topological assessment via embeddings. BMC Bioinf.

[37] Hagberg AA, Schult DA, Swart PJ. Exploring network structure, dynamics, and function using networkx. In: Varoquaux G, Vaught T, Millman J (eds) Proceedings of the 7th python in science conference, Pasadena, CA USA; 2008. p. 11–15

[38] Fey M, Lenssen JE. Fast graph representation learning with PyTorch geometric; 2019

[39] Liben-Nowell D, Kleinberg J. The link prediction problem for social networks. In: Proceedings of the 12th international conference on information and knowledge management. CIKM’03. New York: Association for Computing Machinery; 2003. p. 556–559

[40] Coşkun M, Koyutürk M (2021). Node similarity-based graph convolution for link prediction in biological networks. Bioinformatics.

[41] Alsubait T, Parsia B, Sattler U. Measuring conceptual similarity in ontologies: how bad is a cheap measure? In: Informal Proc. of the 27th international workshop on description logics (DL 2014). CEUR workshop proceedings, vol. 1193. Germany: RWTH Aachen University; 2014. p. 365–377

[42] Dalirsefat SB, Silva Meyer A, Mirhoseini SZ (2009). Comparison of similarity coefficients used for cluster analysis with amplified fragment length polymorphism markers in the silkworm, Bombyx mori. J Insect Sci.

[43] Halu A, De Domenico M, Arenas A, Sharma A (2019). The multiplex network of human diseases. NPJ Syst Biol Appl.

[44] Zhong Y, Li J, He J, Gao Y, Liu J, Wang J, Shang X, Hu J (2020). Twadn: an efficient alignment algorithm based on time warping for pairwise dynamic networks. BMC Bioinf.

[45] Cinaglia P (2024). Gin: a web-application for constructing synthetic datasets of interconnected networks in bioinformatics. SoftwareX.

[46] Nahm FS (2022). Receiver operating characteristic curve: overview and practical use for clinicians. Korean J Anesthesiol.

[47] Parikh R, Mathai A, Parikh S, Chandra Sekhar G, Thomas R (2008). Understanding and using sensitivity, specificity and predictive values. Indian J Ophthalmol.

[48] Retout S, Comets E, Samson A, Mentré F (2007). Design in nonlinear mixed effects models: optimization using the Fedorov–Wynn algorithm and power of the wald test for binary covariates. Stat Med.

[49] Mishra P, Singh U, Pandey CM, Mishra P, Pandey G (2019). Application of student’s t-test, analysis of variance, and covariance. Ann Card Anaesth.

[50] Rosner B, Glynn RJ, Lee M-LT (2006). The wilcoxon signed rank test for paired comparisons of clustered data. Biometrics.

